# Sequence based polymorphic (SBP) marker technology for targeted genomic regions: its application in generating a molecular map of the *Arabidopsis thaliana *genome

**DOI:** 10.1186/1471-2164-13-20

**Published:** 2012-01-13

**Authors:** Binod B Sahu, Rishi Sumit, Subodh K Srivastava, Madan K Bhattacharyya

**Affiliations:** 1 Department of Agronomy, Iowa State University, Ames, Iowa 50011, USA; 2 Molecular, Cellular and Developmental Biology Interdepartmental Graduate Program, Iowa State University, Ames, Iowa 5001, USA

**Keywords:** Niederzenz, Solexa sequencing, sequence based polymorphic marker, nonhost resistance, *Phytophthora sojae*, SHORE analysis

## Abstract

**Background:**

Molecular markers facilitate both genotype identification, essential for modern animal and plant breeding, and the isolation of genes based on their map positions. Advancements in sequencing technology have made possible the identification of single nucleotide polymorphisms (SNPs) for any genomic regions. Here a sequence based polymorphic (SBP) marker technology for generating molecular markers for targeted genomic regions in Arabidopsis is described.

**Results:**

A ~3X genome coverage sequence of the *Arabidopsis thaliana *ecotype, Niederzenz (Nd-0) was obtained by applying Illumina's sequencing by synthesis (Solexa) technology. Comparison of the Nd-0 genome sequence with the assembled Columbia-0 (Col-0) genome sequence identified putative single nucleotide polymorphisms (SNPs) throughout the entire genome. Multiple 75 base pair Nd-0 sequence reads containing SNPs and originating from individual genomic DNA molecules were the basis for developing co-dominant SBP markers. SNPs containing Col-0 sequences, supported by transcript sequences or sequences from multiple BAC clones, were compared to the respective Nd-0 sequences to identify possible restriction endonuclease enzyme site variations. Small amplicons, PCR amplified from both ecotypes, were digested with suitable restriction enzymes and resolved on a gel to reveal the sequence based polymorphisms. By applying this technology, 21 SBP markers for the marker poor regions of the Arabidopsis map representing polymorphisms between Col-0 and Nd-0 ecotypes were generated.

**Conclusions:**

The SBP marker technology described here allowed the development of molecular markers for targeted genomic regions of Arabidopsis. It should facilitate isolation of co-dominant molecular markers for targeted genomic regions of any animal or plant species, whose genomic sequences have been assembled. This technology will particularly facilitate the development of high density molecular marker maps, essential for cloning genes based on their genetic map positions and identifying tightly linked molecular markers for selecting desirable genotypes in animal and plant breeding experiments.

## Background

Discovery of molecular markers has facilitated mapping of both qualitative and quantitative traits. Tightly linked molecular markers facilitate (i) isolation of the genes encoding these traits and (ii) selection of genotypes carrying the desirable alleles. Several molecular marker technologies such as, RFLP, RAPD, DAF, SSR, SSLP, AFLP, CAPS, SNP have been discovered for molecular mapping experiments [1-6]. Fingerprinting of genotypes for restriction fragment length polymorphisms (RFLPs) has been regarded as the most sensitive method of genotyping. This procedure, however, requires a large quantity of genomic DNA and use of radioactive probes. In the random amplified polymorphic DNA (RAPD) marker technology, multiple random loci of the genomes are PCR amplified with a single, 10 nucleotide long primer of arbitrary sequence [3]. In DNA amplification fingerprinting (DAF), many loci are PCR amplified with the aid of a single, short arbitrary primer, as short as 5-nucleotides long [4]. Simple sequence repeat (SSR) markers, also known as microsatellite markers, utilize the variation for tandem repeats such as (CA)_n _repeats observed between genotypes [7]. Simple sequence length polymorphism (SSLP) markers, similar to SSR markers, are designed based on a unique segment of genomic DNA sequence that contains a simple tandem repeat that distinguishes the genotypes. In Arabidopsis, SSLPs are largely based on the (GA)_n _repeats [8]. Cleaved amplified polymorphic sequences (CAPS) markers are designed based on restriction fragment length polymorphisms of PCR amplified fragments, when sequence information of one of the haplotypes is unknown [6].

The high-throughput amplified fragment length polymorphism (AFLP) marker technology combines principles of RFLP and random PCR amplification for rapid identification of molecular loci of the entire genome [1]. AFLP technology is particularly suitable for developing high density molecular marker maps, essential for both map-based cloning of genes and the isolation of molecular markers for selecting desirable genotypes in breeding programs. AFLP technology identifies molecular markers based on a fraction of the restriction fragment length polymorphisms between two genotypes. Restriction site associated DNA (RAD) marker technology, on the other hand, generates markers for all polymorphic sites of a restriction endonuclease between two genotypes; and thus, it is a very sensitive marker technology for developing a high density molecular map [9].

Polymorphisms detected by various marker technologies have been used to generate molecular marker maps of those species that do not have any genome sequences and physical maps. Since assembled genome sequence of many species are available, and the cost of sequencing has declined significantly with advent of the next generation sequencing technologies, single nucleotide polymorphism (SNP) is becoming the most popular molecular marker [10-12]. However, SNP assays are not always simple or flexible. Here, a strategy of using SNPs for rapid generation of molecular markers, termed sequence based polymorphic (SBP) marker technology, is described.

The assembled *Arabidopsis thaliana *genome sequence is selected for this study [13]. Many of the ecotypes of this species are available and have been used in mapping experiments to conduct genetic and biological studies. SNPs among some of the accessions or ecotypes of this model plant species are available [14-15; at http://www.arabidopsis.org]. Niederzenz-0 (Nd-0), used for mapping the *Phytophthora sojae *susceptible (*pss*) mutants that are infected by the soybean pathogen, *P. sojae *(R. Sumit, B.B. Sahu and M.K. Bhattacharyya, unpublished), was selected for this study. The *pss *mutants were created in the *pen1-1 *mutant of the ecotype, Columbia-0 (Col-0). To facilitate mapping of the putative *PSS *gene loci conferring nonhost resistance of Arabidopsis against *P. sojae*, SBP markers were developed as follows. Seventy-five nucleotide long sequencing reads obtained by conducting Solexa sequencing of the Nd-0 genome were compared to Col-0 sequences to identify the SNPs, which were subsequently converted to SBP markers if either of the ecotypes was cut by at least one restriction endonuclease at the SNP sites. By applying this technology, 21 co-dominant SBP markers were generated for the marker-poor regions of the Arabidopsis genome. This novel SBP marker technology should be applicable to any higher eukaryotic species with assembled genome sequences for rapid development of high density molecular marker maps for map-based cloning of genes or identification of suitable molecular markers for selection of desirable genotypes in breeding programs.

## Results

### Generation of a global molecular map for the polymorphic loci of the *Arabidopsis thaliana *ecotypes, Col-0 and Nd-0

Arabidopsis is a nonhost for the soybean pathogen, *Phytophthora sojae*. Several putative *P. sojae *susceptible (*pss*) Arabidopsis mutants that are infected by this oomycete pathogen were identified (R. Sumit, B.B. Sahu and M.K. Bhattacharyya, unpublished). In order to map the putative *PSS *genes that confer nonhost resistance of Arabidopsis against the soybean pathogen, *P. sojae*, a global map of the SSLP and CAPS markers that are polymorphic between ecotypes, Col-0 and Nd-0 was generated. A group of 126 simple sequence length polymorphism (SSLP) markers (http://www.arabidopsis.org) that mapped evenly throughout the entire genome was investigated for polymorphisms. Of these, 50 SSLPs were polymorphic between the two ecotypes. A group of 48 cleaved amplified polymorphic sequences (CAPS) markers also were investigated for polymorphisms between the two ecotypes (Table 1). Of these, 18 were polymorphic between the two ecotypes. The map positions of all 68 polymorphic SSLP and CAPS markers are presented in Additional file 1. Phenotypes of these markers are presented in Additional file 2.

**Table 1:** List of CAPS markers polymorphic between Arabidopsis ecotypes Col-0 and Nd-0

CAPS marker	Restriction enzyme	Primer Sequence
1H1L-1.6	*Rsa*I, *Tsp*509I	F:CTAGAGCTTGAAAGTTGATG R:TTGAGTCCTTCTTGTCTG
20B4L-1.6	*Dde*I	F:CTAAGATGGGAATGTTGG R:GAACTCATTGTATGGACC
40E1T7	*Acc*I	F:GGTCCACTTTGATTCAAGAT R:GCAAGCGATAGAACATAACG
AF2	*Dde*I	F:TCGTCGTTTTTGTTTCCTTTTTCTTA R:CCATTCATTTAGGCCCCGACTTTC
B9-1.8	*Taq*I	F:CATCTGCAACATCTTCCCCAG R:CGTATCCGCATTTCTTCACTGC
CAT2	*Taq*I, *Tsp*509I	F:GACCAGTAAGAGATCCAGATACTGCG R:CACAGTCATGCGACTCAAGACTTG
ER	*Dde*I	F:GAGTTTATTCTGTGCCAAGTCCCTG R:CTAATGTAGTGATCTGCGAGGTAATC
G4711	*Dde*I	F:CCTGTGAAAAACGACGTGCAGTTTC R:ACCAAATCTTCGTGGGGCTCAGCAG
GPA1.4	*Tsp*509I	F:ATTCCTTGGTCTCCATCATC R:GGGATTTGATGAAGGAGAAC
JM411	*Dde*I	F:GCGAACCACTAAGAACTA R:CTCGACTTTGCCAAGGAT
LFY3	*Rsa*I	F:GACGGCGTCTAGAAGATTC R:TAACTTATCGGGCTTCTGC
MI342	*Tsp*509I	F:GAAGTACAGCGGCTCAAAAAGAAG R:TTGCTGCCATGTAATACCTAAGTG
M555	*Acc*I	F:CCTTTAATTAGTTATCAAATC R:CTCTTGAATTATTAAGTTGACTAG
M59	*Rsa*I, *Tsp*509I	F:GTGCATGATATTGATGTACGC R:GAATGACATGAACACTTACACC
MBK23A	*Taq*I	F:GATGATTAGGCGCAAAATTGAG R:ATTACCAGCCTGGCTTCAGG
PAI1.1	*Taq*I, *Rsa*I, *Tsp*509I	F:GATCCTAAGGTATTGATATGATG R:GGTACAATTGATCTTCACTATAG
T20D161	*Taq*I, *Rsa*I, *Tsp*509I	F:CGTATTTGCTGATTCATGAGC R:ATGGTTTACACTTGACAGAGC
T6P5-4.8	*Rsa*I	F:TGAAAGACACCTGGGATAGGC R:CCAACTTTCGGGTCGGTTCC

Restriction endonucleases used for generating individual CAPS markers are shown. F, forward primer; R, reverse primer.

### Generation of SBP markers for saturating a global genome map in *Arabidopsis thaliana*

The global genome map of SSLP and CAPS was marker poor in some genomic regions (Additional file 1). In order to fill out some of the marker poor regions, single nucleotide polymorphism (SNP)-based molecular markers were generated as follows. First, the Nd-0 genome was sequenced in an Illumina/Solexa genome Analyzer II (GAII) at the DNA facility, Iowa State University. Three genome equivalents of Nd-0 sequence in 75 bp reads then were analyzed to discover SNPs (Accession No. SRA048909.1) between Col-0 and Nd-0 by conducting reference guided sequence analysis for all five chromosomes with the aid of the SHORE program [16].

**Figure 1:**
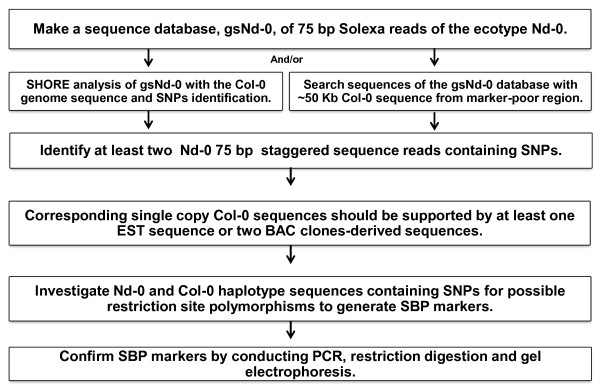
**Steps in generating SBP markers**. Putative SNPs were identified by (i) SHORE mapping of the 75 bp Nd-0 Solexa reads and Co-0 genome sequence and/or (ii) searching SNPs by comparing batches of 50 Kb Col-0 sequences with the 75 bp Nd-0 Solexa reads. The putative SNPs containing Solexa reads that carried staggered ends were selected for next step. Assembled genome sequences of Col-0 carrying putative SNPs were searched for 100% nucleotide matches with transcript sequences or sequences from multiple BACs. The SNPs were utilized to develop SBP markers if they could be translated to restriction fragment length polymorphisms.

**Figure 2:**
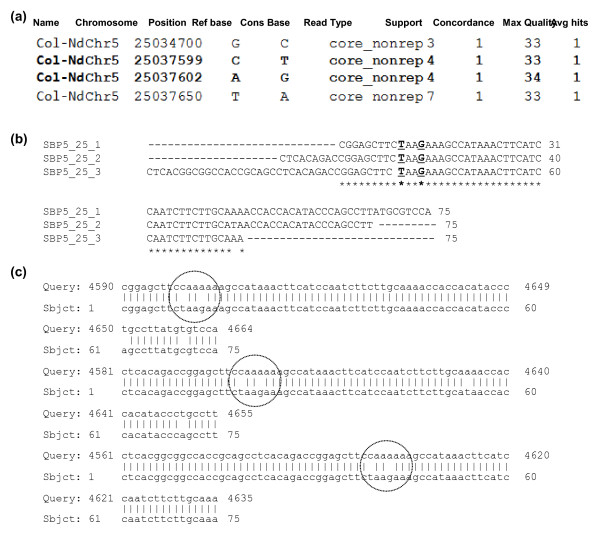
**Identification of SNPs for generation of SBP markers in *Arabidopsis thaliana***. (a) SHORE analysis of a 2.95 Kb DNA fragment of the lower arm of chromosome V between 25,034,700 and 25,037,650 bps resulted in four SNPs. Name, name of the project; Position, position within the chromosome; Ref base, nucleotide of the sequenced genome (Col-0); Cons base, Consensus base (Nd-0); Read type, part of the reads used for prediction were non-repetitive; Support, number of reads supporting a predicted feature; Concordance: Ratio of reads to total coverage of the sequenced genome. Max Quality, highest base quality supporting a prediction; Avg hits, average number of alignments of all reads covering this genomic position. (b) Two SNPs at positions 25,037,599 and 25,037,602 nucleotides [in bold font in (a)] were aligned in three Nd-0 Solexa reads with staggered ends. (c) Three 75 bp Nd-0 Solexa reads were aligned with the reference genome Col-0 (Query Sequence). Two SNPs were circled. Note that the three reads were from three independent DNA molecules.

One can also identify candidate SNPs (NCBI_SS#478443777 through 428555842) for targeted genomic regions by comparing Nd-0 query sequence with Col-0 sequence in batches of ~20 kb (Figure 1). This was achieved by aligning the two sequences using BLAST (bl2seq) program at the NCBI website (http://blast.ncbi.nlm.nih.gov/Blast.cgi). In Solexa sequencing, many sequencing reads could be originated from PCR products of a single DNA molecule (Additional file 3). SNPs originating from 75 bp reads of single PCR molecules are less reliable, because some of such single nucleotide polymorphisms may be generated from PCR-based mutations. This limitation was overcome by selecting those SNPs that originated from at least two staggered 75 bp sequence reads (Figure 2). Staggered reads are considered to originate from independent DNA molecules. Thus, SNPs observed in at least two overlapping reads with staggered ends are considered most likely authentic and selected for the next step. In parallel, to eliminate any possible SNPs originating from sequencing errors in the publicly available Col-0 sequence, the SNPs containing single copy Col-0 sequences were investigated for possible 100% nucleotide matches with (i) expressed sequence tags (ESTs) or (ii) genomic sequences of at least two BACs (Additional file 4) in GenBank. The Col-0 sequences that met one of these criteria were considered further for SBP marker development. Use of the above two criteria in selecting SNP-containing sequences increased the chance of SBP marker identification. High quality SNPs identified through SHORE analysis could be directly applied for developing SBP markers, if genomes are sequenced to higher depth (≥ 20X genome equivalents).

**Figure 3:**
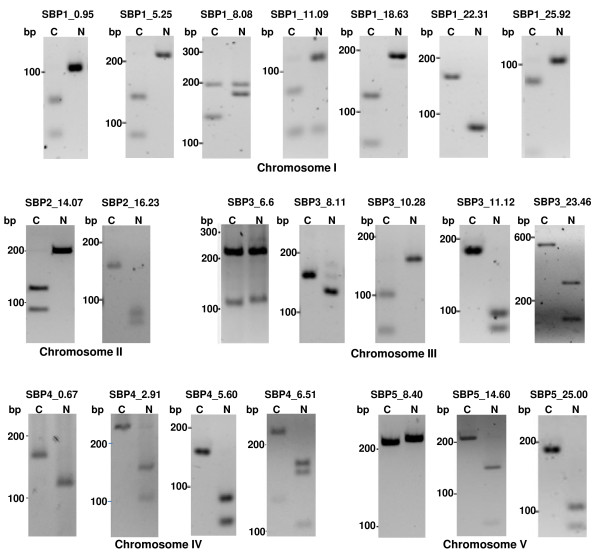
**SBP markers generated to fill out the marker poor regions of the genetic map developed based on polymorphisms between the ecotypes Col-0 and Nd-0**. Primers for PCR amplification and restriction enzymes used in generating the SBP markers are listed in Table 2.

**Figure 4:**
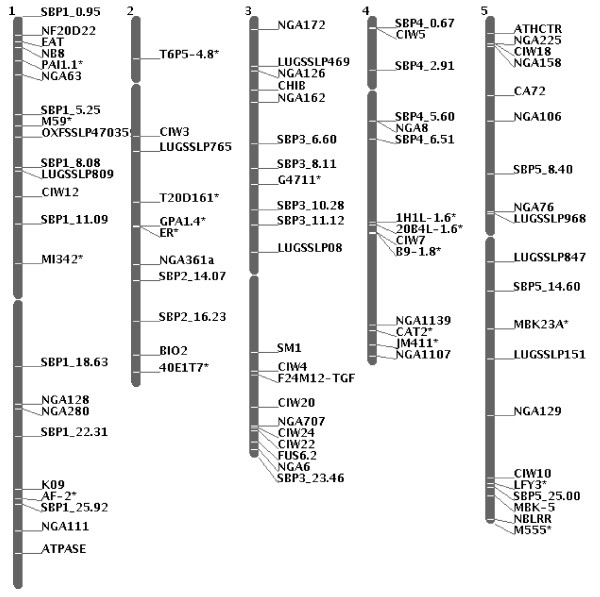
**The molecular map of the five Arabidopsis chromosomes showing the locations of the SBP markers**. Primers for 18 CAPS and 21 SBP markers are listed in Tables 1 and 2, respectively. Primer sequences for the 50 SSLP markers can be obtained from the TAIR database. CAPS markers are distinguished from SSLP markers with asterisks.

In the last step of the SBP marker development, SNPs were converted to possible restriction endonuclease site-specific polymorphisms between the Col-0 and Nd-0 haplotypes by analyzing restriction enzyme digestion patterns of the selected Nd-0 and Col-0 sequences using a suitable program (http://tools.neb.com/NEBcutter2/). PCR amplicons of approximately 200 nucleotides and that contained variations for restriction endonuclease sites between Col-0 and Nd-0 ecotypes were considered as putative SBP markers. Finally, primers for PCR amplification were designed in such a way that one can easily distinguish the haplotype-specific restriction enzyme length polymorphisms following separation of the restriction enzyme digested PCR products on a 4% (w/v) agarose gel. Following this protocol, 21 SBP markers for some of the marker poor regions of the Arabidopsis genome were identified (Figures 3 and 4; Table 2).

**Table 2:** Primers and restriction enzymes used in generating 21 SBP markers

**Ch**. **No**.	Name	Primer sequence	Restriction enzyme	Amplicon size (bp)
1	SBP1_0.95	F:GTCAGGCTAGCTCATCAAGTCCTAC R:CCGGATTTCGCCAGCTCCGTC	*Tsp*509I	100
1	SBP1_5.25	F:CACAAACCCTTCACCTCCAT R:GCAGTTGCCTAAAGGCTGAG	*Msp*I	233
1	SBP1_8.08	F:AACGCAATTCTCAAGCAGGT R:CATTCAATTGTTGGGCAGTG	*Rsa*I	188
1	SBP1_11.09	F:AAAGTCAACCGGGAGGTTTC R:AGGCTGAGGACACGAGAAGA	*Bam*HI	163
1	SBP1_18.63	F:GCACTTGCAAAAGGAAGCTC R:TTCTTGCTGGAGAATCGTGA	*Rsa*I	197
1	SBP1_22.30	F:TACCGGTTCCGGTCACTATC R:AATGGGAAATTGGGATTGGT	*Dde*I	151
1	SBP1_25.92	F:TTGTTGAGAGAGCGAGATCAAA R:AAAAGCATCACATCATCTTTGG	*Nhe*I	102
2	SBP2_14.07	F:GAAGGAATTGGACCAAACGA R:ATCTAGCTGCCCTCACTGGA	*Bts*CI	213
2	SBP2_16.23	F:CACCATTTGTTCCCGTAAGC R:TGGTCAATCCATGGTGATGT	*Hpy*166II	157
3	SBP3_6.60	F: CCATCGTCCTATTCTAATCCATGTTG R: GATGCAAAATCTCCATCCTCTTC	*Tsp*509I	379
3	SBP3_8.11	F:CACGTATCGGCGAGTCTACA R:CAAATTCAAATCTCAGTTTTCGTC	*Taq*I	150
3	SBP3_10.28	F:TCTAAAACGAACCGGGAAAA R:CGACAAGTAAATTAAAACCAACCTG	*Mbo*II	151
3	SBP3_11.12	F:AAGACTTTGGTTCAACTCCTGAA R:GGCTTTGGATTCAGGAAAAA	*Taq*I	184
3	SBP3_23.46	F: CGACCAAATGTCTCTGAGATGTTC R: CACCCAAGGCGGTGTTGGCGAAAG	*Taq*I	520
4	SBP4_0.67	F:CGGTTAACATGCCTCAATCC R:TGTGGATGATTTGGGGACTC	*Dde*I	171
4	SBP4_2.91	F:CGAGTGACTTCTTGAGGTTTATTATG R:CGAGATTGCTTTGGTATGGA	*Hpy*166II	249
4	SBP4_5.60	F:AGGGAAGAATATGCGGAAGG R:TGTTTCTGTCTTGGCCCATT	*Taq*I	159
4	SBP4_6.51	F:GGACAAGACCTTGATTTGAAGTTTG R:GAGGGCTCACATTGGGTTTAATG	*Tsp*509I	395(C), 490(N)
5	SBP5_8.40	F:TCGACGGTGACTTGTAGGTG R:CGATGCCGTCTCATAAAAGG	*Dpn*I	232
5	SBP5_14.60	F: CGCGGTTATGGTAACGTTAAATG R: CCGAGGGAGAAGAAAGGATCAAGAAG	*Hph*I	225
5	SBP5_25.00	F:AAATCACCAATGGCAAAACA R:TTTGCGTAGACGGAGAGTGA	*Dde*I	191

Restriction endonucleases used for generating individual SBP markers are shown. F, forward primer; R, reverse primer; C, Col-0; N, Nd-0.

## Discussion

The use of molecular markers has gained importance in genetic studies particularly for map based cloning of genes [17]. The relatively low cost of sequencing a genome, with the emergence of high throughput sequencing technology, has facilitated genome wide polymorphism studies [18,19]. The SBP marker technology can convert most of the single nucleotide polymorphisms to molecular markers for any genomic regions. SBP markers developed based on sequence information are ideal for those species, whose genomes are sequenced and assembled. Reference genome sequence can be utilized to develop SBP markers for a specific genomic region with known physical location. Thus, marker-poor regions can be enriched with SBP markers. In this study, the applicability of the SBP marker technology for generating markers is shown for improving a genetic map that represents polymorphisms between two Arabidopsis ecotypes, Col-0 and Nd-0 (Figure 4). SBP markers were generated from just three genome equivalents Nd-0 genome sequence of 75 bp Solexa reads. The method also has been successfully applied in developing a high density molecular map of the *PSS1 *gene that confers nonhost resistance against the soybean pathogens, *Phytophthora sojae *and *Fusarium virguliforme *(R. Sumit, B.B. Sahu and M.K. Bhattacharyya, unpublished).

The SHORE program used in this study is highly powerful and has been employed successfully in identification of a mutation through analysis of deep sequence data of a bulk of 500 mutant F_2 _progenies [20]. If the genome sequencing is not conducted to a higher depth (e.g. ≥ 20 fold), SNPs identified through SHORE analyses can be verified by conducting BLAST analyses. Staggered Solexa sequence reads (Figure 2) containing SNPs are considered for generating SBP markers for such a scenario. Similarly, candidate SNP containing regions of the reference genome should be supported by multiple sequences, such as transcript sequences and/or sequences from more than one BAC clone to avoid any possible sequencing errors (Additional file 4).

If none of the haplotypes of interest are sequenced, then reference genome sequence should be used to define the SNP maps of individual haplotypes by running the SHORE program. The SNP maps then can be compared to determine the SNPs between the haplotypes of interest. Once the candidate SNPs are identified, small PCR amplicons of ~ 200 bp can be amplified and digested with suitable restriction endonuceases to release the restriction length polymorphisms. A significant proportion of the SNPs could be unusable in SBP marker development because they may not be digested with restriction endonucleases in a haplotype- or genotype-specific manner. In such a case, one can apply derived CAPS (dCAPS) technology to improve the efficiency of SBP marker development [21].

## Conclusions

A new molecular marker technology, based on genome sequence and physical map locations, is reported for those species whose assembled genome sequences are available. The technology was applied in identifying 21 SBP markers for some of the marker-poor genomic regions of the Arabidopsis molecular marker map that represent polymorphisms between ecotypes, Col-0 and Nd-0 (Figure 4). The SBP marker technology should be applicable to any genomic regions and will facilitate (i) map-based cloning genes as well as (ii) the development of tightly linked molecular markers for selecting desirable genotypes in animal and plant breeding experiments.

Ease in SBP marker development and application to any genomic regions, and genome-wide abundance of SNPs make this technology suitable for mapping experiments, especially to develop high density molecular maps for positional gene cloning experiments, if the assembled genome sequence and physical maps of the studied species are available. Innumerable SBP markers can be developed rapidly for a genomic region containing a target gene in a map-based cloning experiment. Co-dominant gel-based SBP markers are ideal to identify genetic recombination events between two loci. Such PCR-based markers can be used to screen a large number of segregants to identify informative recombinants of the target gene region. These recombinants will then facilitate the development of high resolution maps of a large number of SBP markers, essential for cloning genes based on their map position. Thus, high-throughput deep sequencing, together with SBP markers, should expedite map-based cloning in higher eukaryotes.

## Methods

### Plant materials and growth conditions

Seeds of *Arabidopsis thaliana *ecotypes, Col-0 and Nd-0, were sown on LC1 soil-less mixture (Sun Gro Horticulture, Bellevue, WA) under 16 h light/8 h dark regime at 21°C with approximately 60% relative humidity. The light intensity was maintained at 120-150 μE/m^2^/s [22]. Ten days after sowing, the seedlings were transplanted in LC1 mixture. The newly transplanted seedlings were covered with humidity domes for two days and thereafter watered every fourth day. A fertilizer mixture of 15:15:15::N:P:K (1% concentration v/v) was applied to the seedlings seven days after transplantation.

### DNA preparation and the whole genome sequencing

Genomic DNA was extracted from Arabidopsis by the CTAB method [23]. Either young inflorescence or a rosette leaf was selected for DNA extraction. The Nd-0 genome was sequenced in a Solexa, Illumina sequencing platform at the DNA facility, Iowa State University. The 75 bp Solexa Nd-0 reads were saved as the gsNd database (Accession No. SRA048909.1) for further studies.

### Analysis of the raw reads from Solexa Sequencing

The raw 75 bp Solexa reads of the gsNd database were analyzed by the mapping algorithms, Efficient Large scale Alignment of Nucleotide Databases (ELAND), which is built in with the Solexa sequence analysis pipeline of the Illumina sequencer [24]. This program can match a large number of reads against a reference genome sequence; e.g., in this study the Arabidopsis Col-0 genome sequence was used as the reference genome. In order to identify the SNPs from the entire Arabidopsis genome (NCBI_SS#478443777 through 428555842), the 75 bp Solexa sequence reads of Nd-0 were compared to the assembled Col-0 genome sequence (version TAIR10) (ftp://ftp.arabidopsis.org/home/tair/Sequences/whole_chromosomes/) by running the SHORE program [25]. The gsNd database also was used for conducting the BLASTN (bl2seq) search for polymorphic sequences of the marker poor genomic regions.

### SSLP and CAPS markers polymorphic between Col-0 and Nd-0

Candidate SSLP and CAPS markers available from the TAIR database were selected to cover the entire genome. Sequence information of primers for SSLP markers were obtained from Bell and Ecker [9] and the Arabidopsis Information Resource (TAIR) database (http://www.arabidopsis.org). The chromosome map tool function available at the TAIR database (http://www.arabidopsis.org/jsp/ChromosomeMap/tool.jsp) was used to map the physical locations of the markers that showed polymorphisms between the two accessions.

### PCR conditions and digestion with restriction endonucleases

The final DNA concentration in PCR was 20 ng/μl. The PCR mixtures contained 2 mM MgCl_2 _**(**Bioline, Taunton, MA), 0.25 μM each of forward and reverse primer, 2 μM dNTPs and 0.5 U Choice Taq polymerase (Denville Scientific, Inc., Metuchen, NJ). For SBP or SSLP, PCR was conducted at 94°C for 2 min, and then 40 cycles of 94°C for 30 s, 50°C or 55°C for 30 s and 72°C for 30 s. Finally, the mixture was incubated at 72°C for 10 min. For CAPS markers, PCR was conducted at 94°C for 2 min, and then five cycles of 94°C for 30 s followed by decreasing annealing temperatures from 55°C to 50°C (-1°C/cycle) and 72°C for 1 min. Then 35 cycles of 94°C for 30 s, 50°C for 30 s, and 72°C for 1 min were conducted. Finally, the reaction mixtures were incubated at 72°C for 10 minutes. PCR was carried out in PTC-100 Programmable Thermal Controllers (MJ Research Inc., Waltham, MA). The amplified products were resolved on a 4% (w/v) agarose gel at 8 V/cm. Amplified CAPS and SBP products were digested with the respective restriction enzymes following manufacturer's protocols. The ethidium bromide stained PCR products were visualized by illuminating with UV light.

## Authors' contributions

MKB conceived and designed the experiments. SKS conducted bioinformatics analyses and identified sequence reads for selected regions of the genome. BBS and RS analyzed the sequence data, performed experiments and analyzed experimental data. BBS and MKB wrote the manuscript. All authors read and approved the final manuscript.

## Supplementary Material

Additional file 1 **Arabidopsis molecular genome map generated based on SSLP and CAPS markers that are polymorphic between Col-0 and Nd-0 ecotypes**. CAPS markers shown with asterisks. The map was drawn using the chromosome map tool available at TAIR. Click here for file

Additional file 2 **Phenotypes of the SSLP and CAPS markers polymorphic between Col-0 and Nd-0 ecotypes**. C, Col-0; N, Nd-0. Click here for file

Additional file 3 **Two 75 bp Nd-0 Solexa reads most likely originated from a single DNA molecule**. The two reads showed similarity in their identity to a specific Col-0 sequence. Most likely the two sequence reads were obtained from sequencing of two molecules generated through PCR of a single DNA molecule. Click here for file

Additional file 4 **The Col-0 sequence carrying SNPs, shown in Figure 2 (a), showed identity to three cDNA sequences**. Click here for file
