# Supplementary figures and images for: Sequence based polymorphic (SBP) marker technology for targeted genomic regions: its application in generating a molecular map of the Arabidopsis thaliana genome
Source: BMC Genomics. 2012 Jan 13;13:20. doi: 10.1186/1471-2164-13-20 (PMC3323429; doi:10.1186/1471-2164-13-20)

## Slide 1
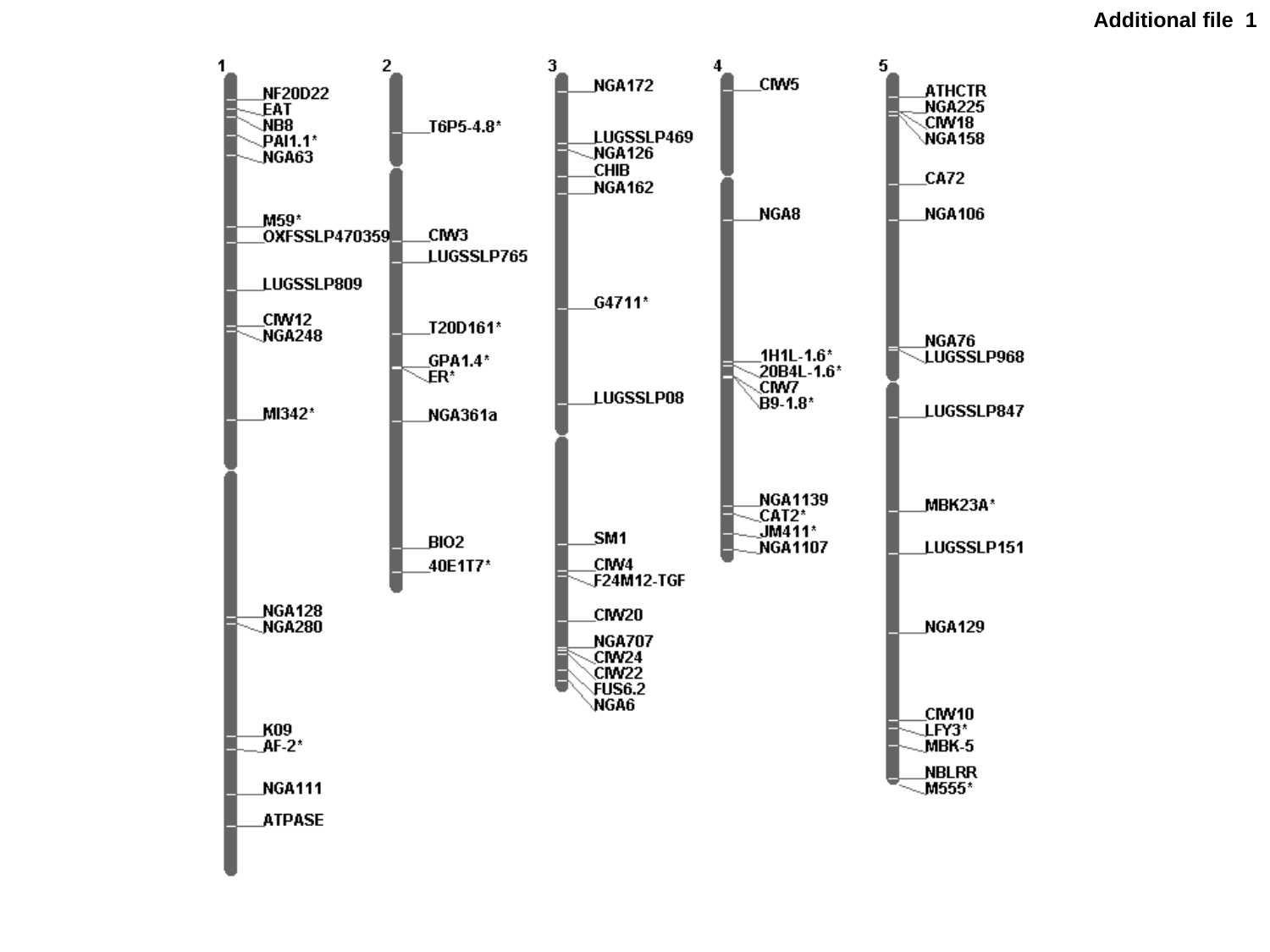

Additional file 1

Supplement: Additional file 1 — Arabidopsis molecular genome map generated based on SSLP and CAPS markers that are polymorphic between Col-0 and Nd-0 ecotypes. CAPS markers shown with asterisks. The map was drawn using the chromosome map tool available at TAIR. [file 1471-2164-13-20-S1.PPTX]

## Slide 1
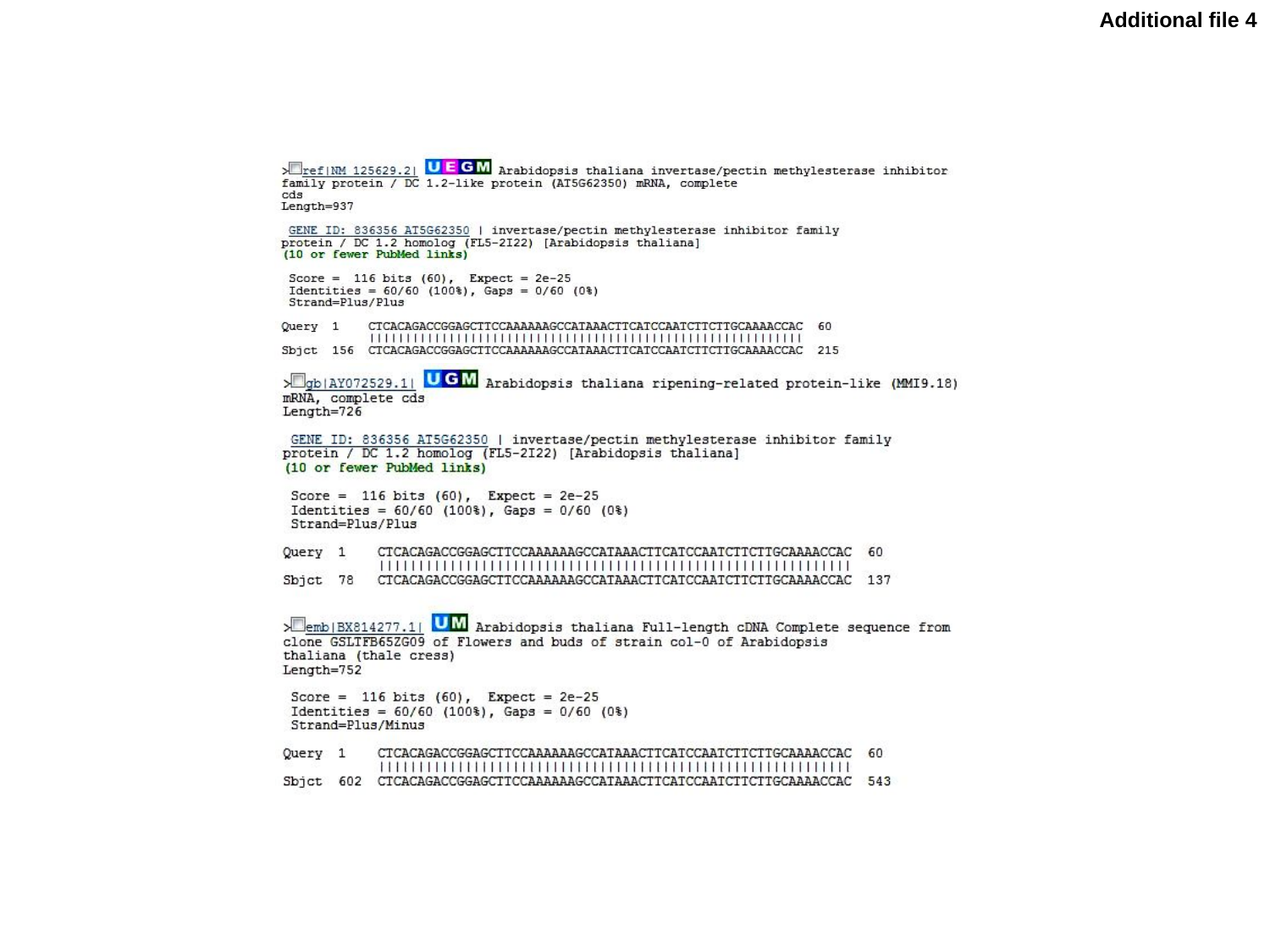

Additional file 4

Supplement: Additional file 4 — The Col-0 sequence carrying SNPs, shown in Figure 2 (a), showed identity to three cDNA sequences. [file 1471-2164-13-20-S4.PPTX]
