# Supplementary material for: Sequence based polymorphic (SBP) marker technology for targeted genomic regions: its application in generating a molecular map of the Arabidopsis thaliana genome
Source: BMC Genomics. 2012 Jan 13;13:20. doi: 10.1186/1471-2164-13-20 (PMC3323429; doi:10.1186/1471-2164-13-20)
Supplement: Additional file 2 — Phenotypes of the SSLP and CAPS markers polymorphic between Col-0 and Nd-0 ecotypes. C, Col-0; N, Nd-0. [file 1471-2164-13-20-S2.PPTX]

## Slide 1
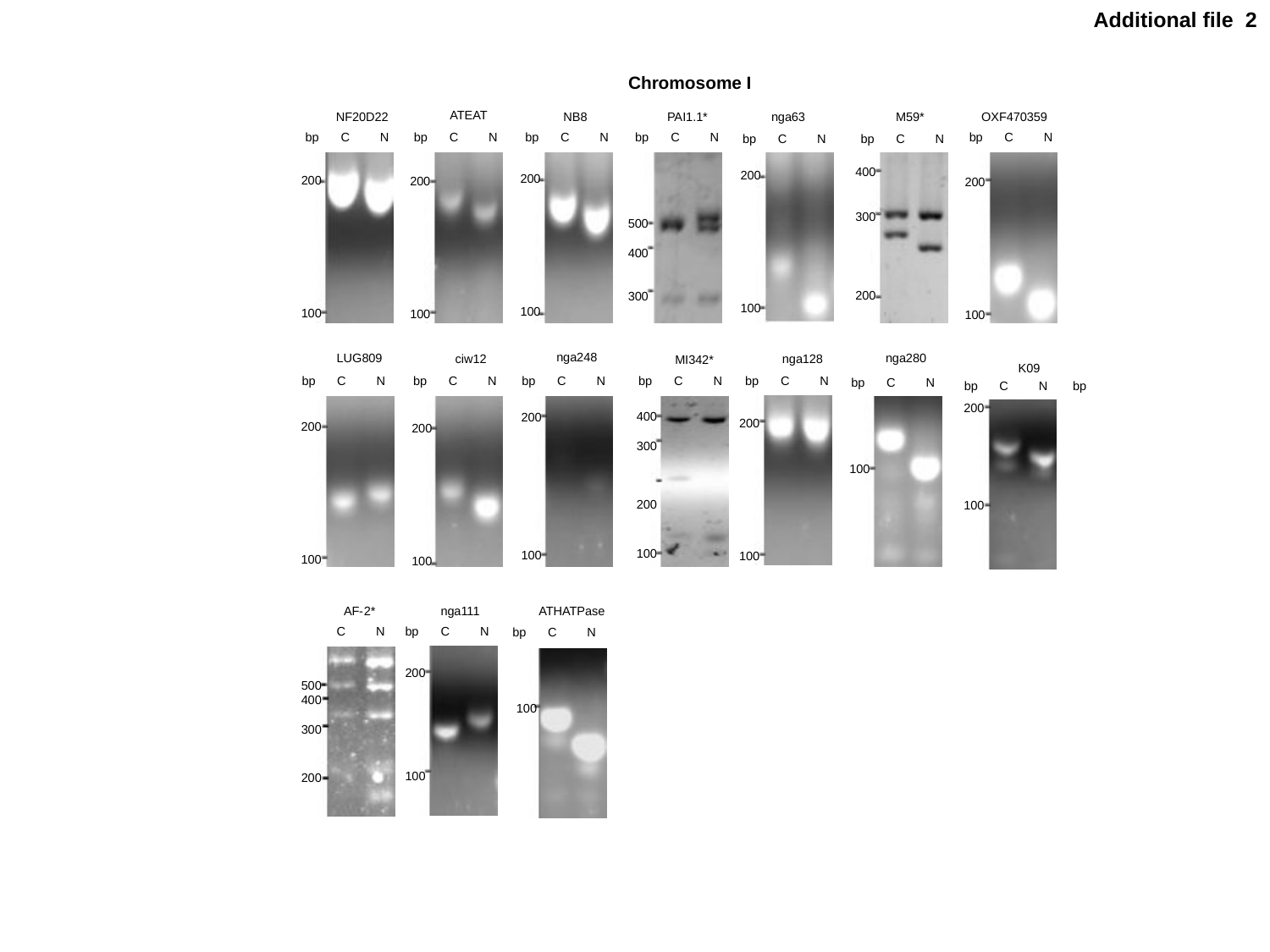

Additional file 2
Chromosome I
ATEAT
NF20D22
NB8
PAI1.1*
nga63
M59*
OXF470359
bp
C N
bp
C N
bp
C N
bp
C N
bp
C N
bp
C N
bp
C N
400
200
200
200
200
200
300
500
400
200
300
100
100
100
100
100
nga248
nga280
LUG809
ciw12
nga128
MI342*
bp
C N
bp
C N
bp
C N
bp
C N
bp
C N
bp
C N
400
200
200
200
200
300
100
200
100
100
100
100
100
K09
bp
C N
bp
200
100
AF
-
2*
ATHATPase
nga111
C N
bp
C N
bp
C N
200
500
400
100
300
100
200

## Slide 2
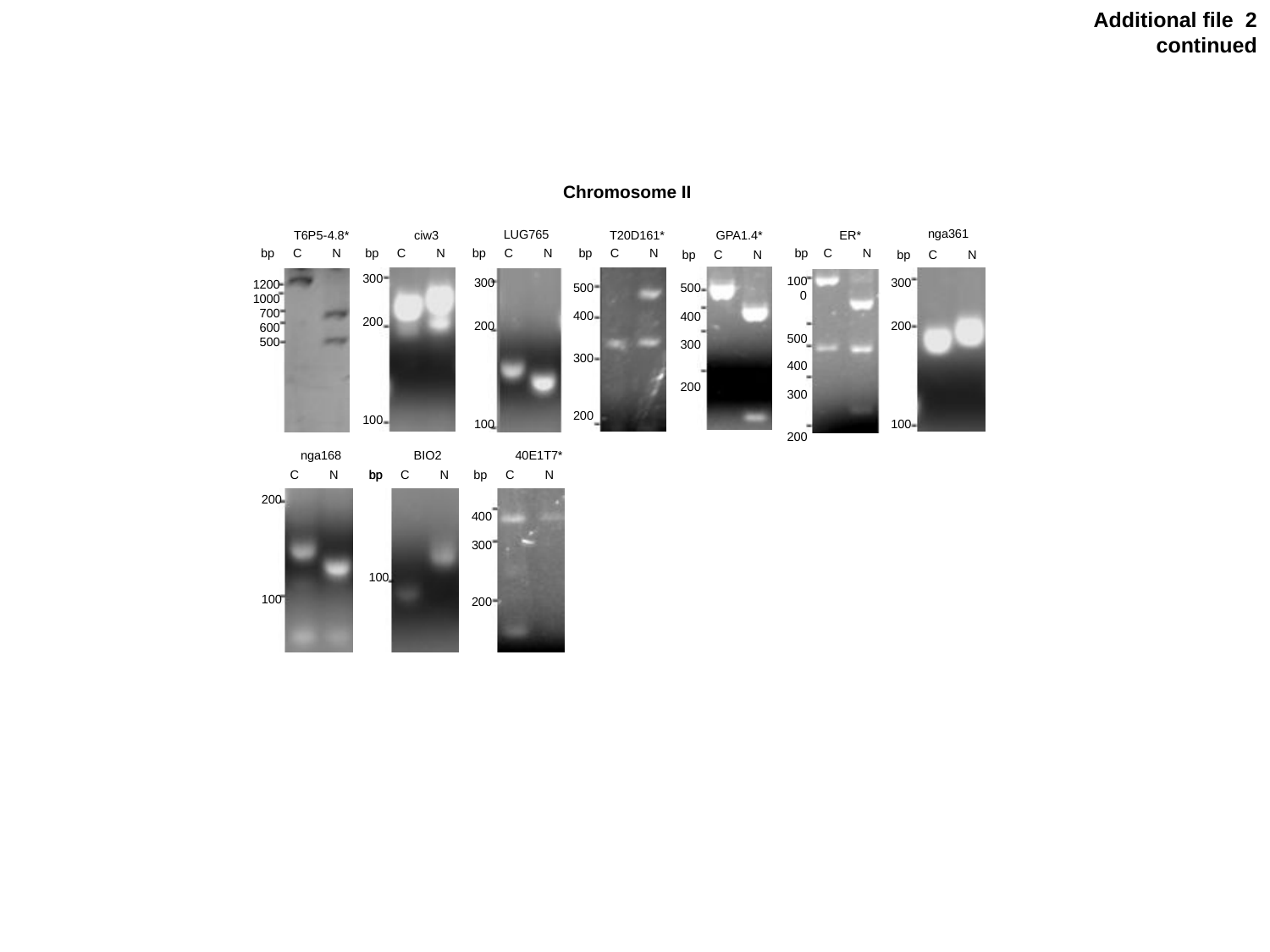

Additional file 2
continued
Chromosome II
nga361
LUG765
T6P5
-
4.8*
ciw3
T20D161*
GPA1.4*
ER*
bp
C N
bp
C N
bp
C N
bp
C N
bp
C N
bp
C N
bp
C N
300
100
300
300
1200
500
500
0
1000
700
400
400
200
200
200
600
500
500
300
300
400
200
300
200
100
100
100
200
nga168
BIO2
40E1T7*
C N
bp
bp
C N
bp
C N
200
400
300
100
100
200

## Slide 3
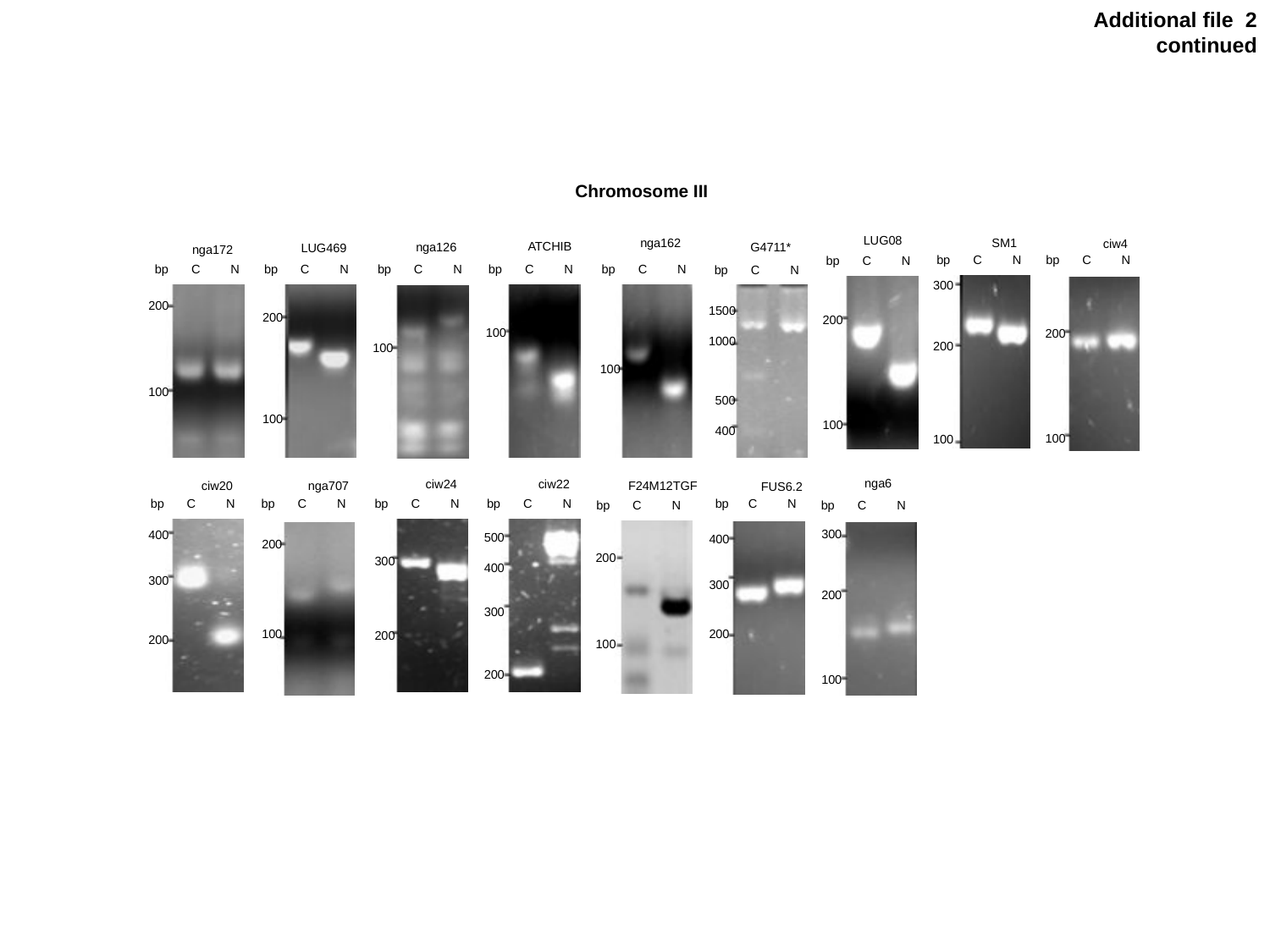

Additional file 2
continued
Chromosome III
LUG08
nga162
ATCHIB
G4711*
nga126
LUG469
nga172
bp
C N
bp
C N
bp
C N
bp
C N
bp
C N
bp
C N
bp
C N
200
1500
200
200
100
1000
100
100
100
500
100
100
400
SM1
ciw4
bp
C N
bp
C N
300
200
200
100
100
nga6
ciw24
ciw22
nga707
ciw20
F24M12TGF
FUS6.2
bp
C N
bp
C N
bp
C N
bp
C N
bp
C N
bp
C N
bp
C N
300
400
500
400
200
200
300
400
300
300
200
300
100
200
200
200
100
200
100

## Slide 4
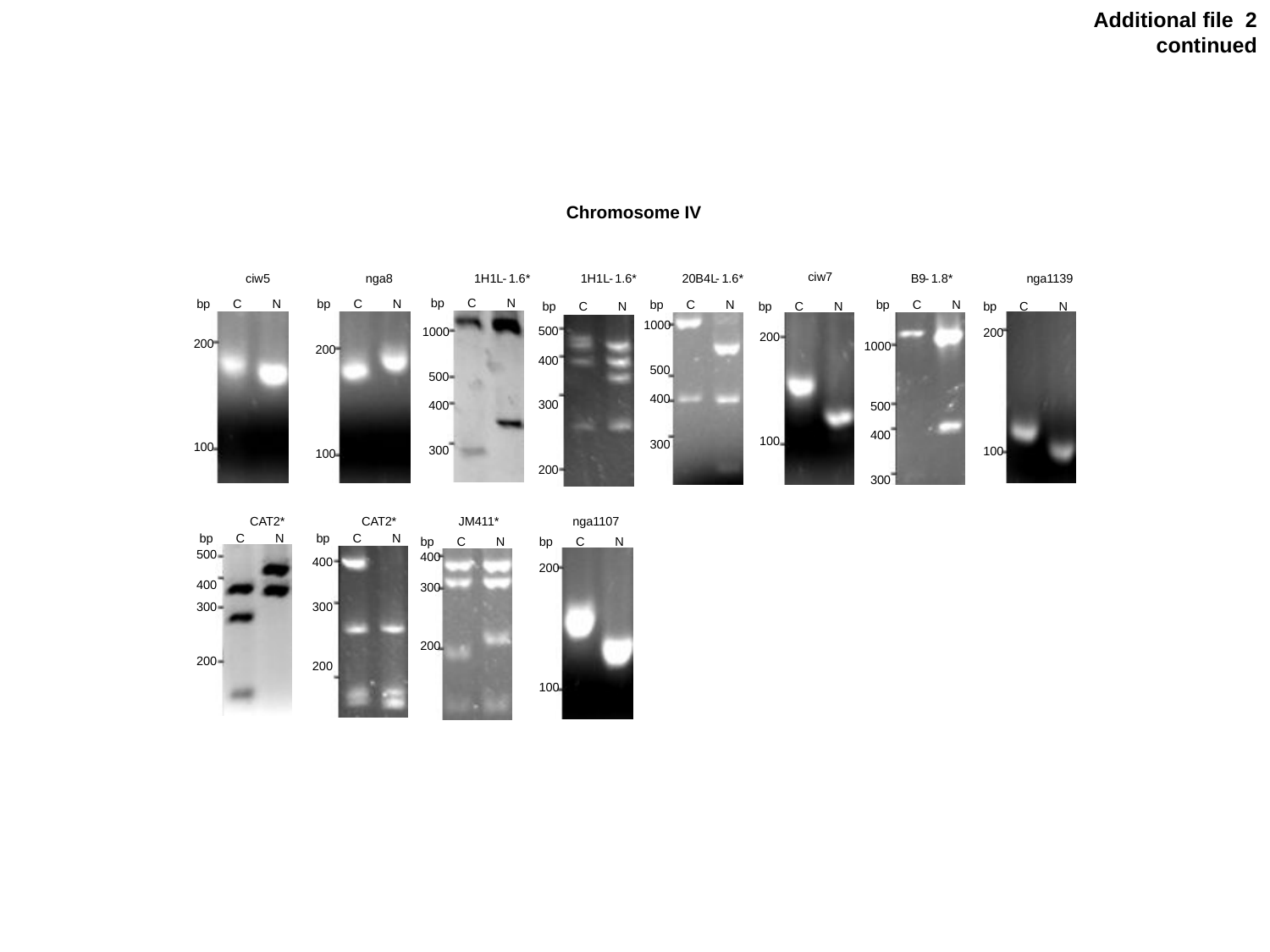

Additional file 2
continued
Chromosome IV
ciw7
ciw5
nga8
1H1L
-
1.6*
1H1L
-
1.6*
20B4L
-
1.6*
nga1139
B9
-
1.8*
bp
C N
bp
C N
bp
C N
bp
C N
bp
C N
bp
C N
bp
C N
bp
C N
1000
500
1000
200
200
200
1000
200
400
500
500
400
300
400
500
400
100
300
100
300
100
100
200
300
CAT2*
CAT2*
JM411*
nga1107
bp
C N
bp
C N
bp
C N
bp
C N
500
400
400
200
400
300
300
300
200
200
200
100

## Slide 5
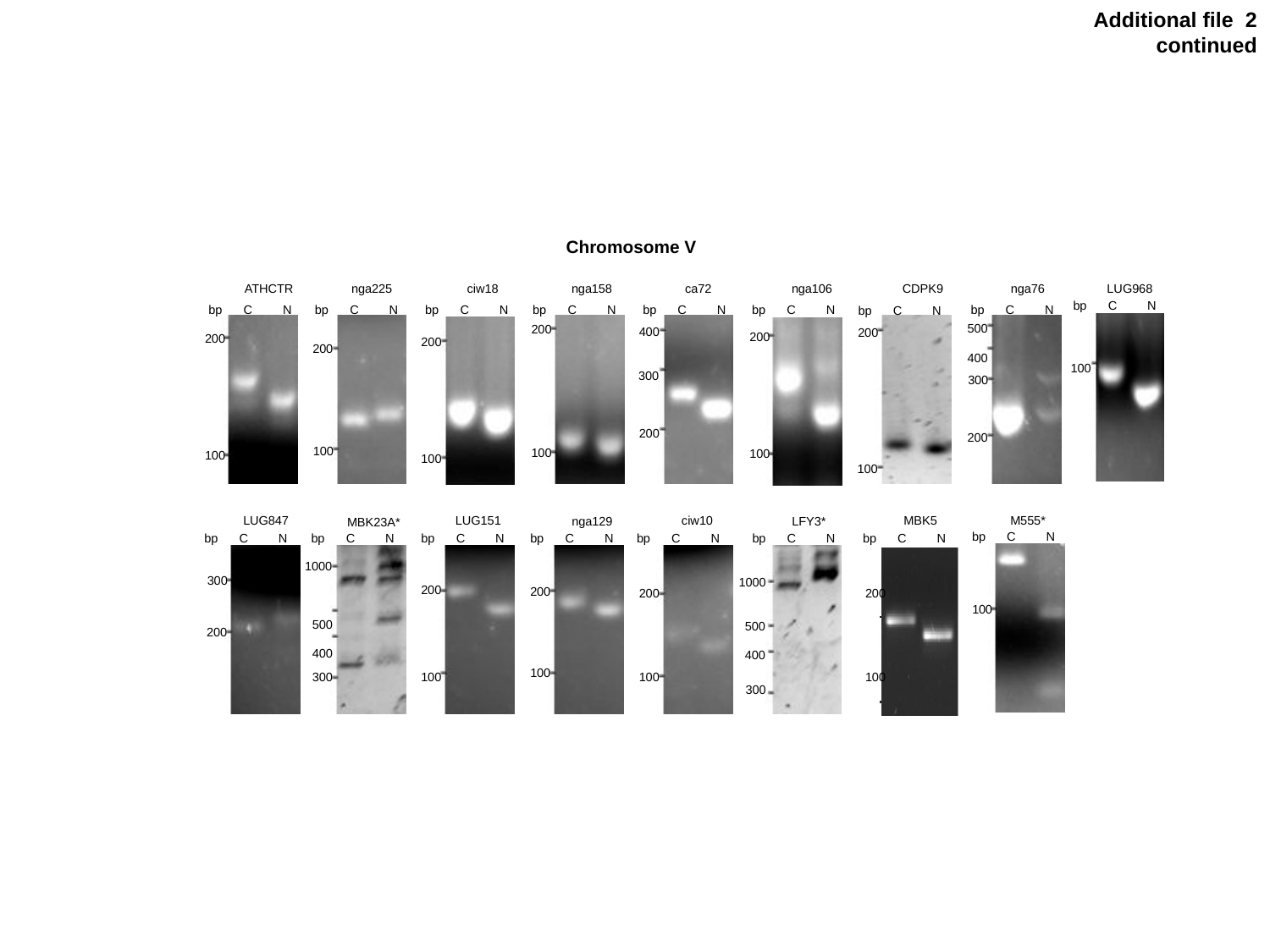

Additional file 2
continued
Chromosome V
ATHCTR
nga225
ciw18
nga158
ca72
nga106
CDPK9
bp
C N
bp
C N
bp
C N
bp
C N
bp
C N
bp
C N
bp
C N
200
400
200
200
200
200
200
300
200
100
100
100
100
100
100
nga76
bp
C N
500
400
300
200
LUG968
bp
C N
100
ciw10
LUG847
LUG151
nga129
LFY3*
MBK23A*
bp
C N
bp
C N
bp
C N
bp
C N
bp
C N
bp
C N
1000
300
1000
200
200
200
500
500
200
400
400
100
100
300
100
300
MBK5
bp
C N
200
100
M555*
bp
C N
1
00
