# Supplementary material for: Sequence based polymorphic (SBP) marker technology for targeted genomic regions: its application in generating a molecular map of the Arabidopsis thaliana genome
Source: BMC Genomics. 2012 Jan 13;13:20. doi: 10.1186/1471-2164-13-20 (PMC3323429; doi:10.1186/1471-2164-13-20)
Supplement: Additional file 3 — Two 75 bp Nd-0 Solexa reads most likely originated from a single DNA molecule. The two reads showed similarity in their identity to a specific Col-0 sequence. Most likely the two sequence reads were obtained from sequencing of two molecules generated through PCR of a single DNA molecule. [file 1471-2164-13-20-S3.PPTX]

## Slide 1
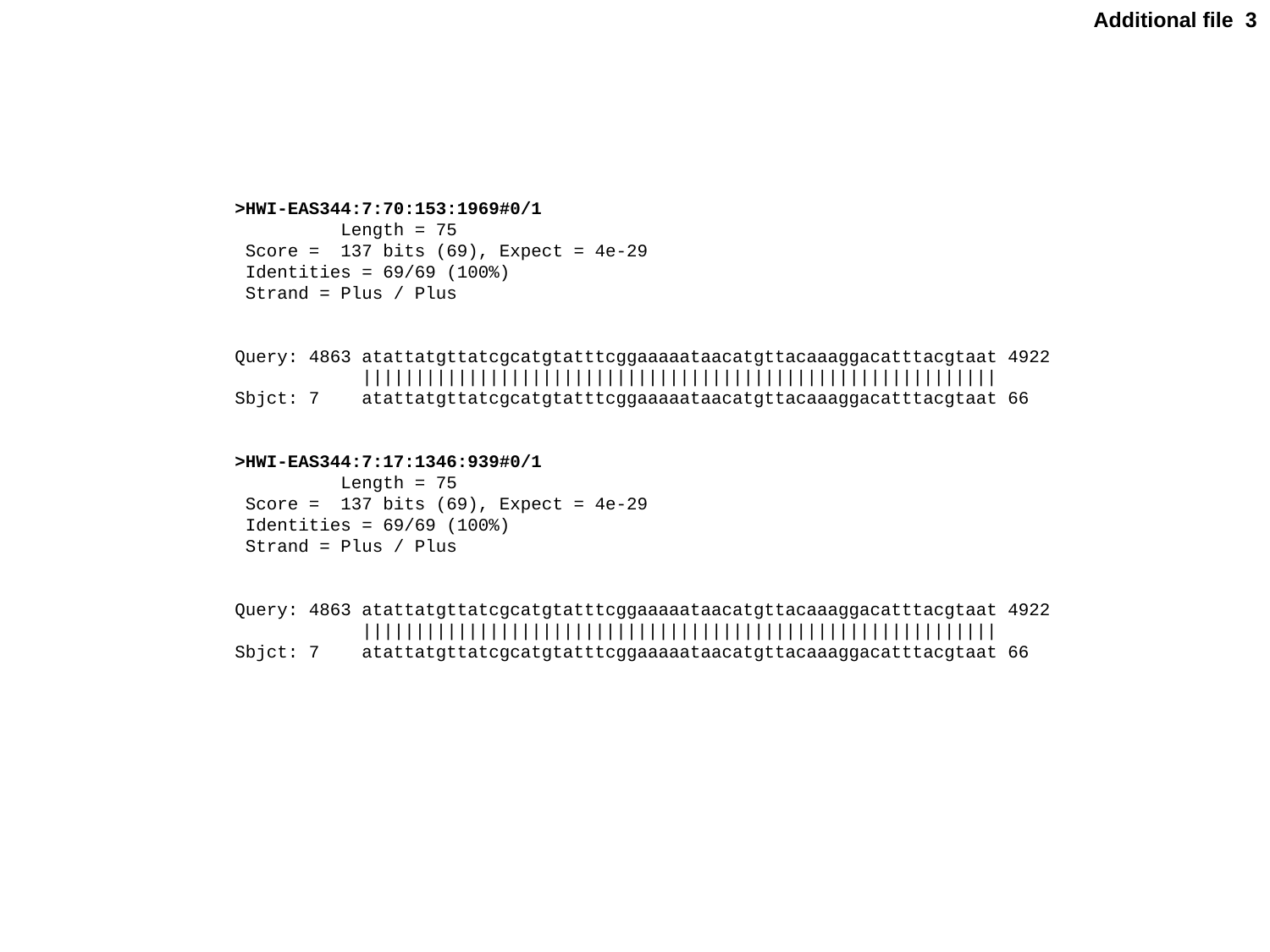

Additional file 3
>HWI-EAS344:7:70:153:1969#0/1
 Length = 75
 Score = 137 bits (69), Expect = 4e-29
 Identities = 69/69 (100%)
 Strand = Plus / Plus
Query: 4863 atattatgttatcgcatgtatttcggaaaaataacatgttacaaaggacatttacgtaat 4922
 ||||||||||||||||||||||||||||||||||||||||||||||||||||||||||||
Sbjct: 7 atattatgttatcgcatgtatttcggaaaaataacatgttacaaaggacatttacgtaat 66
>HWI-EAS344:7:17:1346:939#0/1
 Length = 75
 Score = 137 bits (69), Expect = 4e-29
 Identities = 69/69 (100%)
 Strand = Plus / Plus
Query: 4863 atattatgttatcgcatgtatttcggaaaaataacatgttacaaaggacatttacgtaat 4922
 ||||||||||||||||||||||||||||||||||||||||||||||||||||||||||||
Sbjct: 7 atattatgttatcgcatgtatttcggaaaaataacatgttacaaaggacatttacgtaat 66
